# Comparative analysis of atmospheric radiative transfer models using the Atmospheric Look-up table Generator (ALG) toolbox (version 2.0)

**DOI:** 10.5194/gmd-13-1945-2020

**Published:** 2020-04-20

**Authors:** Jorge Vicent, Jochem Verrelst, Neus Sabater, Luis Alonso, Juan Pablo Rivera-Caicedo, Luca Martino, Jordi Muñoz-Marí, José Moreno

**Affiliations:** 1 Magellium, Toulouse, France; 2 Image Processing Laboratory, Universitat de València, 46980 Paterna, Valencia, Spain; 3 Finnish Meteorological Institute, Erik Palménin aukio 1, 00560 Helsinki, Finland; 4 Secretary of Research and Graduate Studies, CONACYT-UAN, 63155 Tepic, Nayarit, Mexico; 5 Departamento de Teoría de la Señal y Comunicaciones, Universidad Rey Juan Carlos, 28943 Fuenlabrada, Madrid, Spain

## Abstract

Atmospheric radiative transfer models (RTMs) are software tools that help researchers in understanding the radiative processes occurring in the Earth’s atmosphere. Given their importance in remote sensing applications, the intercomparison of atmospheric RTMs is therefore one of the main tasks used to evaluate model performance and identify the characteristics that differ between models. This can be a tedious tasks that requires good knowledge of the model inputs/outputs and the generation of large databases of consistent simulations. With the evolution of these software tools, their increase in complexity bears implications for their use in practical applications and model intercomparison. Existing RTM-specific graphical user interfaces are not optimized for performing intercomparison studies of a wide variety of atmospheric RTMs. In this paper, we present the *Atmospheric Look-up table Generator* (ALG) version 2.0, a new software tool that facilitates generating large databases for a variety of atmospheric RTMs. ALG facilitates consistent and intuitive user interaction to enable the running of model executions and storing of RTM data for any spectral configuration in the optical domain. We demonstrate the utility of ALG in performing intercomparison studies of radiance simulations from broadly used atmospheric RTMs (6SV, MODTRAN, and libRadtran) through global sensitivity analysis. We expect that providing ALG to the research community will facilitate the usage of atmospheric RTMs to a wide range of applications in Earth observation.

## 1 Introduction

Atmospheric radiative transfer models (RTMs) have deeply helped in understanding the radiation processes occurring in the Earth’s atmosphere (Dubovik and King, 2000; Iacono et al., 2008). RTMs are physically based computer models that numerically describe the absorption, emission, and scattering processes in the ultraviolet to microwave region. Therefore, they are widely used in Earth observation scientific and technological applications, such as (i) sensor/mission design (Kerekes et al., 1999; Verhoef and Bach, 2012; Verstraete et al., 2015), (ii) atmospheric chemistry (Theys et al., 2007; Dubovik et al., 2011), (iii) meteorology and climatology (Forster et al., 2011), (iv) atmospheric correction (Richter, 1996; Cooley et al., 2002; North et al.,2008), and (v) atmospheric physics (Stamnes et al., 1988). Over time and through continuous improvements, these models have increased in realism from simple semiparametric equations (e.g., Seidel et al., 2010) towards advanced RTMs that allow for explicit 3D representations of complex interactions in the atmosphere. Some examples include, 6SV (Vermote et al., 1997), libRadtran (Mayer and Kylling, 2005; Emde et al., 2016), MODTRAN (Berk et al., 2006, 2014), MOMO (Fell and Fischer, 2001), and RTTOV (Saunders et al., 2018).

Given the importance of atmospheric RTMs for remote sensing applications, their intercomparison is one of the main tasks used in order to determine their performance and to identify the characteristics that differ between models (Kotchenova et al., 2008; Seidel et al., 2010; Proud et al.,2010; Callieco and Dell’Acqua, 2011). The process of comparing various atmospheric RTMs can be a tedious task that requires good knowledge of the model inputs/outputs and the generation of large databases of consistent simulations. Indeed, the evolution of RTMs towards more advanced models has resulted in an increase in complexity and interpretability of these models, which bears implications for practical implementation of intercomparison studies. To overcome this limitation, graphical user interfaces (GUIs) have been developed to facilitate RTM use and execution. A few examples of these GUIs can be found for 6SV (Matarrese et al., 2015; Wilson, 2013), MODTRAN (Schläpfer, 2016; Berk et al.,2017), or libRadtran (Mayer and Kylling, 2017). These well-documented tools allow complete access to all functionalities and configuration parameters of the models they were designed for, including user support and continuous updates. However, each of these GUIs are customized for their specific RTM; and none can be used to define and run simulations for multiple RTMs in a consistent manner. In addition, they are not designed to easily precompute large databases, which are important due to the high computational burden of performing statistical analysis (Verrelst et al., 2016) or running these models in a pixel-per-pixel basis (Gastellu-Etchegorry et al., 2003; Guanter et al., 2009). Altogether, these GUIs are not fully offering practical solutions for the implementation of atmospheric RTMs in Earth observation applications and, in particular, for model intercomparison. Users of atmospheric RTMs are therefore obliged to develop their own specific scripts to create datasets, which are typically (1) limited to a handful input variables and (2) hardly extensible to other RTMs.

In an attempt to facilitate the consistent simulation of databases for a wide range of atmospheric RTMs, we developed the *Atmospheric Look-up table Generator* (ALG). ALG is a MATLAB-compiled software package that allows generating look-up tables (LUTs) based on a suite of atmospheric RTMs. Namely, a LUT consists of a collection of input atmospheric conditions and corresponding generated RTM spectral outputs (see Sect. 3.3 for further details). ALG provides consistent and intuitive user interaction for defining model configuration and running and storing RTM data for any spectral configuration in the optical domain. The main objectives of this paper are therefore to (1) describe the ALG tool from a functional and software design perspective, thereby giving the reader an overview of the implemented features and generated LUT data; and (2) perform a comparison study between the models implemented in ALG: MODTRAN (v5 and v6), 6SV v2.1, and libRadtran v2.0.2.

The remainder of this work is structured as follows: Sect. 2 gives an overview of the currently implemented atmospheric RTMs and associated graphical interfaces. Section 3 describes the ALG software design and its main features. Section 4 provides a comparative analysis of the implemented atmospheric RTMs. Section 5 summarizes a few applications as examples of the usage of ALG. Finally, Sect. 6 concludes with an outlook of on-going and planned functionalities to be implemented in future versions of ALG.

## 2 Overview of existing atmospheric RTMs and associated GUIs

In this section we describe the key features of the atmospheric RTMs compatible with ALG version 2.0 and their user interfaces.

### 2.1 Modtran

Developed by Spectral Science Inc. (http://modtran.spectral.com/, last access: 15 April 2020), MODTRAN (Berk et al.,2006, 2014) is one of the most widely used RTMs by scientists and commercial organizations with multiple applications in Earth observation. MODTRAN solves the atmospheric radiative transfer (RT) equation with the discrete ordinates (DISORT) method (Stamnes et al., 1988) and a statistical simulation of the absorption effects through the correlated-*k* method (Goody et al., 1989). The coupled absorption and scattering simulations are calculated in a stratified spherically symmetric atmosphere composed of vertical profiles of molecules (e.g., Anderson et al., 1986). Suspended particles (aerosols) are divided into the boundary layer (< 2 km) and stratosphere. Accordingly, MODTRAN combines the effects of molecular and particulate absorption/emission and scattering, surface reflections/emission, solar/lunar illumination, and spherical refraction. The spectral outputs include direct and diffuse transmittance, top-of-atmosphere (TOA) radiance fluxes, solar/lunar irradiance, horizontal fluxes, cooling rates, etc. These outputs cover the 0.2–200 μm spectral range and are provided at a resolution of up to 0.1 cm^–1^ (1–100 pm in the VIS-SWIR spectral range) for the narrow band simulations or higher using line-by-line capabilities (Berk and Hawes, 2017). With over 30 years of heritage, MODTRAN has been extensively validated and continues to be maintained and upgraded (Berk et al., 2015).

Several GUIs are commercially available by companies such as Spectral Sciences Inc., Ontar’s PcModWin (https://ontar.com/pcmodwin-6, last access: 15 April 2020), and ReSe’s MODO (Schläpfer, 2016). These tools consist of a graphical front-end that wraps around MODTRAN, facilitating user interaction and model configuration from scratch, thus leveraging the use of MODTRAN. These GUIs give access to a wide range of input parameters definition such as vertical profiles, geometric conditions and spectral configuration. Users can therefore format the input files to run MODTRAN and display the output simulations through interactive plotting panels. Some of these tools also allow the running of several simulations, manually varying the configuration of every new simulation or through a parameter series of one variable at a time. Despite these capabilities, none of these tools are customized to generate large LUTs.

### 2.2 6SV

6S was developed in the 90s (Vermote et al., 1997). Since then, it has been applied to process broadband resolution instruments (e.g., El Hajj et al., 2008; Matthews et al., 2010; Liu et al., 2012). 6S solves the RT equation based on the method of successive orders of scattering (Lenoble, 1985), with a decoupling of the absorption and scattering effects of molecules and particulates. These numerical approximations are performed in a stratified plane-parallel atmosphere composed of vertical profiles of molecules and aerosols. An exponential vertical profile is used for the aerosol concentration, and the optical properties are assumed to be the same in the entire atmospheric column. The calculated spectral outputs include direct and diffuse transmittance in the sun-to-target and target-to-sensor directions, spherical albedo, atmospheric path radiance, and TOA radiance fluxes. These outputs extend from the spectral range between 0.3 and 4 μm at a resolution of 2.5 nm. The latest updates of the code account for polarization in the atmosphere (Kotchenova et al., 2006; Kotchenova and Vermote, 2007).

The only GUI dedicated to 6S known by the authors is its official website (http://6s.ltdri.org/, last access: 15 April 2020). Under its section “Run 6SV”, users can define the input configuration and run the code to retrieve the 6S input and output files directly from the web browser. Accordingly, the generation of multiparametric LUTs is not feasible with this online GUI. In order to overcome this limitation, Py6S was developed (Wilson, 2013). Py6S is a Python-based application programming interface that provides (1) a user-friendly model setting, (2) run and plotting capabilities, and (3) the ability to import external data (e.g., atmospheric profiles). As such, Py6S can be integrated in any Python code facilitating the direct usage of 6S in data processing algorithms or for LUT generation.

### 2.3 LibRadtran

The libRadtran software package is a collection of algorithms for atmospheric radiative transfer calculations (http://www.libradtran.org/doku.php, last access: 15 April 2020) and thus used for various applications in the field of remote sensing, atmospheric physics, and climatology. LibRadtran implements different solvers of the RT equation (DISORT among them) that allow computing (polarized) radiances, irradiances, and actinic fluxes in the solar and thermal spectral regions with a resolution of up to 1 cm^–1^ (0.01–0.6 nm in the VIS-SWIR spectral range) (Mayer and Kylling, 2005;Emde et al., 2016). LibRadtran is a user-friendly RTM that, similar to MODTRAN, allows users to define and configure the atmospheric state with a wide variety of options, including molecules, aerosols water/ice clouds, and surface boundary conditions. The most recent updates include new features such as a (1) simulation of the Raman scattering, (2) new parameterization of molecular absorption called Reptran (Gasteiger et al., 2014) and aerosol optical properties, or (3) Monte Carlo solver of the RT equation. The flexible design of libRadtran makes it a powerful and versatile tool for research tasks. Furthermore, libRadtran includes a Python-based graphical user interface that simplifies the usage of the model. The GUI has similar functionalities to those previously discussed for MODTRAN. As such, it is not possible to run a large set of simulations and compile LUTs for later use in data processing applications.

### 2.4 OPAC

The OPAC package is a widely used software tool that provides aerosol optical properties in the 0.25 and 40 μm spectral range (Hess et al., 1998; Koepke et al., 2015). OPAC calculates the extinction, scattering, and absorption coefficients, the single scattering albedo, the asymmetry parameter, and the phase function. These optical properties are calculated for a set of 10 predefined aerosol models and user-defined mixtures, thus expanding the existing capabilities of atmospheric RTMs.

Similar to the previously defined RTMs, OPAC operates on the basis of input/output files. In order to facilitate its use, several GUIs have been developed that are compatible with OPAC. MOSPMAP is a toolbox, linked with libRadtran, for the optical modeling of complex aerosols, including precalculated optical properties of single aerosol particles as those in the OPAC package (Gasteiger and Wiegner, 2018). A userfriendly web interface was developed for MOPSMAP facilitating online calculations. The AEROgui tool (Pedrós et al.,2014) is a similar GUI package that can be used to obtain the optical properties of a mixture of aerosol particles. Accordingly, AEROgui expands the current capabilities of OPAC by also providing a user interface to facilitate user definition of new aerosol mixtures. However, in most cases, there is no direct and straightforward way to include the OPAC output data into atmospheric RTM simulations.

## 3 The Atmospheric Look-up table Generator (ALG) tool

In this section we identify the key software functionalities (Sect. 3.1). Then we introduce the ALG interface and how it is used to configure a new LUT (Sect. 3.2). Finally, we describe how ALG automatically generates a LUT and its content (Sect. 3.3).

### 3.1 Key software functionalities

The primary goal of ALG is to provide a scientific software package that fills the gaps observed in the previously analyzed tools. In particular, (1) each existing GUI is compatible with only one specific atmospheric RTM (e.g., PcModWin for MODTRAN) and cannot be used to configure and run simulations for other RTMs, (2) these tools are not intended to run a large number of simulations and thus to create LUTs, and (3) the inputs and outputs of each atmospheric RTM are generally not consistent between each other, adding an extra layer of complexity when using or comparing various models. Accordingly, ALG is designed to offer the following key functionalities:

1. ALG functions as a wrapper for running atmospheric RTMs, providing a graphical tool in which users can select the input configuration (i.e., atmospheric, geometric, and spectral). In this way, ALG keeps the same functionality as all the previously described tools.
2. ALG facilitates the integration of additional atmospheric RTMs. In its current version 2.0, ALG is compatible with MODTRAN5 (Berk et al., 2006), MODTRAN6 (Berk et al., 2014), 6SV version 2.1 (Vermote et al., 1997), and libRadtran version 2.0.2 (Emde et al.,2016).
3. The GUI is common to all the implemented atmospheric models, facilitating their configuration and execution.
4. LUT design with ALG is a flexible process in which users can select a RTM and its input atmospheric variables and values.
5. ALG automatically processes and harmonizes all the RTM input and output data into the final LUT file. With this functionality, ALG facilitates the intercomparability between atmospheric RTMs and the possibility to alternate between models in a data processing algorithm (e.g., for atmospheric correction).
6. ALG provides a help system and a set of tutorials to facilitate users with the installation and operation of the software.

### 3.2 ALG graphical interface

ALG’s graphical interface provides users the tools to configure the software, to run the RTM simulations, and to construct the final LUT. It is divided into three main elements: the *Software configuration*, the *LUT configuration*, and the *Help system* GUIs.

The *Software configuration* GUI facilitates the user to edit software aspects of ALG such as (1) the path to the executable RTM files, (2) the default folder to store the output data, and (3) the default CPU cores used to run a RTM. In addition, users can add new RTM input variables and edit their default values. This software configuration GUI also permits editing and storing of the spectral configuration of existing and user-defined remote sensing instruments to ease the generation of sensor-specific LUTs.

**Figure 1:**
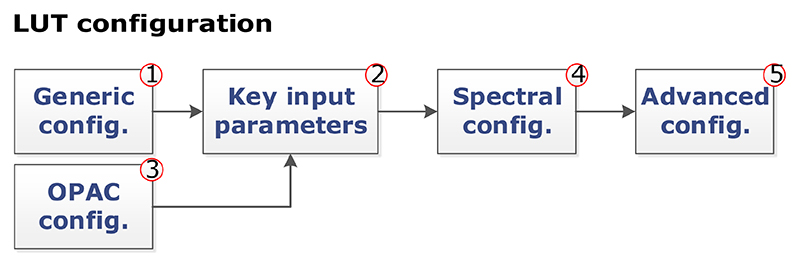
LUT configuration steps accessible through ALG’s graphical interface.

In its core interface (*LUT configuration*) users can select the RTM input variables and values used to run the simulations and to store the spectral outputs into the LUT. This GUI is based on the commonalities found in Sect. 2, with extended functionalities that allow the running of a large set of simulations. The LUT configuration GUI is divided into five main subsequent steps as shown in Fig. 1 and further described in the paragraphs below.

In step 1 (*Generic configuration*), the atmospheric RTM used to run the simulation and the sampling method used to distribute the LUT nodes (i.e., collection of points of input atmospheric and geometric variables) are selected. Several methods are implemented to distribute LUT nodes, including the following: (a) systematic gridded combinations of all input values, typically applied in atmospheric correction algorithms (e.g., Guanter et al., 2009); (b) scattered nearrandom and homogeneous sampling of the input variable space based on Latin hypercube sampling (McKay et al.,1979), Sobol distribution (Bratley and Fox, 1988), and Halton distribution (Kocis and Whiten, 1988); or (c) automatic gradient-based distribution (Vicent et al., 2018). Parallel instances of the selected atmospheric RTM are invoked in order to speed up the process of generating large LUTs (Brazile et al., 2008).

**Figure 2:**
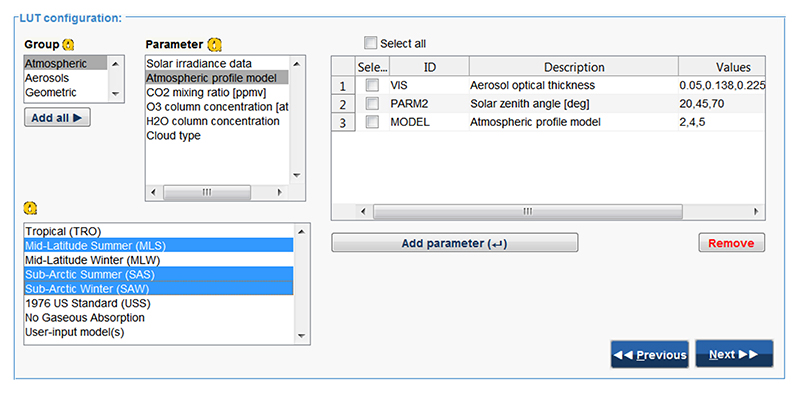
The *key input parameters* of the LUT configuration GUI (see step 2 in Fig. 1) allows users to introduce input model variables and their values.

In step 2 (*Key input parameters*), ALG allows users to introduce selected atmospheric and geometric variables and their values (see Fig. 2). In ALG, input variables are divided into two types: discrete and continuous. Discrete variables are those that can only take on a certain number of values. Typical examples of discrete variables are the atmospheric profile, the aerosol model, or the extraterrestrial solar irradiance. Continuous variables can have any value within an allowed range. Typical examples of continuous variables are the columnar water vapor (CWV), the aerosol optical thickness (AOT), or the solar/viewing zenith angle (SZA/VZA). For continuous variables, their values vary between an userinput minium/maximum range and, in case of gridded sampling, are distributed according to a selected distribution (linear, logarithmic, exponential, or cosine).

In step 3 (*OPAC configuration*), ALG implements a backend interface with OPAC (v3.1) database, expanding the predefined aerosol models with a comprehensive database of aerosol optical properties (i.e., extinction, absorption, and phase function). For OPAC aerosol models, users can create new aerosol mixtures described by their particle number density from a set of basic components.

In step 4 (*Spectral configuration*), the spectral configuration of the RTM simulations is introduced. Users can set the desired spectral range and resolution, eventually at noncontiguous spectral intervals, saving computation time and disk storage of unwanted wavelengths. A set of predefined spectral configurations of common satellite instruments or user-defined sensors can be loaded.

Finally, in step 5 (*Advanced configuration*), the user has access to advanced RTM configuration parameters (e.g., selection of radiative transfer solver, printed output files). These parameters largely depend on the selected RTM.

All these configuration parameters are stored in an .xml file that is later used by ALG’s internal functions (see Sect. 3.3) to automatically run the RTM simulations and construct the final LUT. This configuration file can be loaded by ALG, allowing users to edit and rerun previous simulations, e.g., by adding new atmospheric variables, changing the spectral configuration or modifying advanced settings. It worth also noticing that the LUT configuration interface is common for all implemented RTMs, and the software harmonizes the naming and definition of atmospheric and geometric parameters to all models.

Additionally, ALG’s GUI provides access to the help system with information about (1) how to install the software and third-party RTMs, (2) how to generate a new LUT, (3) sample cases (tutorials) with practical applications of the use of the software, and (4) implemented RTMs and input variables. The ALG help system is based on MATLAB^®^ help browser developed by © The MathWorks, Inc.

### 3.3 ALG internal functions. Look-up table generation

**Figure 3:**
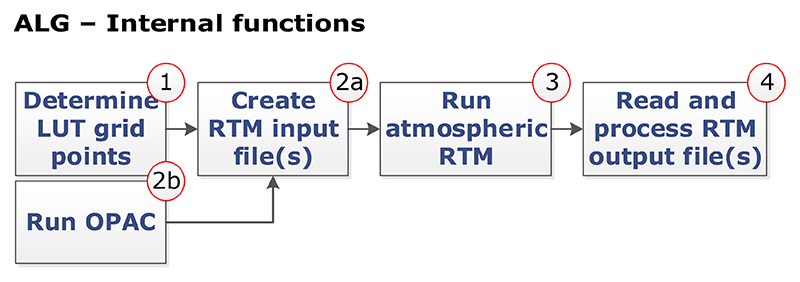
ALG’s internal functions for RTM model execution and LUT generation process.

After setting the LUT configuration (see Sect. 3.2), ALG implements a set of backend functionalities to automatically generate the output atmospheric LUT based on the input configuration (see Fig. 3).

In step 1, ALG starts determining the LUT nodes of input atmospheric and geometric variables according to the selected option. Three LUT node distribution methods are implemented in ALG. The first method corresponds to a systematic (gridded) combination of all input variables and their values. Assuming *D* selected input variables, each of them with *p_i_* values (*i =* 1 to *D*), the output LUT will contain $N=\overset{D}{\underset{\mathrm{i=1}}{\prod^{\text{​}}}}p_{i}$ nodes. The second method corresponds to a pseudorandom distribution of nodes homogeneously covering the *D*-dimensional input space with the user-defined *N* scattered nodes. The final method is based on an automatic node distribution algorithm, GALGA, that minimizes the error in the linear interpolation of simulated TOA radiance below a user-defined error threshold value. This gradient-based node distribution has been shown to reduce interpolation errors by at least 10 % and LUT size by at least 25 %(Vicent et al., 2018). ALG includes a multidimensional interpolation function that works both with gridded and scattered data. The implemented LUT interpolation methods involve (1) nearest neighbor, (2) piecewise linear (Abramowitz and Stegun, 1964), (3) piecewise cubic splines (Bartels and Barsky, 1998), (4) inverse distance weighting (Shepard, 1968), and (5) *D*-dimensional triangulation (Delaunay, 1934; Barber et al., 1996).

In step 2a, the LUT generation process continues by converting the determined combinations of atmospheric/geometric variables and user-input spectral configuration into a set of RTM input files required to build the atmospheric LUT. In this step, ALG detects if the user has selected any default or user-defined OPAC aerosol model. If so, ALG automatically runs OPAC and saves the output aerosol optical properties for a later use (see step 2b). Following the approach proposed in Huang et al. (2016), the values of these aerosol properties, spectral configuration, and additional atmospheric input variables (i.e., the LUT nodes) are written in *P* subsets of RTM input files. In step 3 (Run *atmospheric RTM*), parallel instances of the selected RTM are then run in batch mode based on these input files.

In step 4, and once all the RTM simulations are correctly executed, ALG will finalize the LUT generation process by reading, processing, and storing the RTM output data files in the final LUT file. One of the key aspects of ALG is that it harmonizes the variety of RTM spectral outputs into a common and consistent definition of the stored LUT data. For this, ALG uses the so-called *atmospheric transfer functions*, typically used in remote sensing applications. These atmospheric transfer functions permit uncoupling of the radiative transfer effects the between the surface and atmosphere and thus are particularly useful in atmospheric correction and forward modeling (Vermote et al., 1997; Matthew et al., 2000; Guanter et al., 2009; Verhoef and Bach, 2012). In the case of a Lambertian and homogeneous surface with reflectance *ρ*, a TOA radiance spectrum (***L***_toa_) can be calculated through Eq. (1):

(1) $${\text{L}}_{\text{toa }}={\text{L}}_{0}+\frac{\left({\text{E}}_{\text{dir}}\mu_{\text{il}}+{\text{E}}_{\text{dif}}\right)\left({\text{T}}_{\text{dir}}+{\text{T}}_{\text{dif}}\right)\text{ρ}}{\pi (1-\text{S}\rho )}.$$

where *μ*_il_ is the cosine of the SZA. The LUTs generated by ALG contain the atmospheric transfer functions used in Eq. (1) and which are described below:

– The spectrum of intrinsically reflected radiance by the Earth’s atmosphere (***L***_0_; mWm^–2^sr^–1^nm^–1^), also called atmospheric path radiance.
– The downwelling solar irradiance spectrum at surface level, split by its direct (***E***_dir_) and diffuse (***E***_dif_) fluxes, both in milliwatts per square meter per nanometer (mWm^–2^nm^–1^).
– The atmospheric reflectance spectrum for the photons backscattered to the surface (***S***), also known as spherical albedo.
– The upwelling direct and diffuse target-to-sensor transmittance spectra (***T***_dir_ and ***T***_dif_).

In addition to these atmospheric transfer functions, the generated LUT file also includes:

– The extraterrestrial solar irradiance spectrum at 1AU Earth-to-Sun distance, ***I***_0_ in milliwatts per square meter per nanometer (mW m^–2^ nm^–1^).
– The wavelength vector at which these spectral magnitudes are calculated.
– The name and values of the input atmospheric and geometric variables for each LUT node.
– The values of the remaining (constant) parameters.

An important part of the complexity of ALG lies in being able to harmonize the different radiative transfer codes, with different types of outputs, to fill the exact same LUT. For MODTRAN simulations, these spectrally dependent atmospheric transfer functions are automatically calculated by applying the interrogation technique presented in Guanter et al. (2009) and Verhoef and Bach (2012). In the case of libRadtran simulations, four runs are needed to compute these transfer functions (Debaecker et al., 2016). Similarly, 6SV directly provides the atmospheric transfer functions, however, with a slightly different definition due to the uncoupling of scattering and gas transmittance. The following transfer functions are used for 6SV: path radiance, at-surface total solar irradiance due to scattering (***E***_tot_; mWm^–2^nm^–1^), total gas transmittance (***T***_gas_), total upwelling transmittance due to scattering (***T***_tot_), and spherical albedo (***S***). In this case, ***L***_toa_ is calculated through Eq. (2):

(2) $${\text{L}}_{\text{toa }}={\text{L}}_{0}+\frac{{\text{T}}_{\text{gas }}{\text{E}}_{\text{tot }}{\text{T}}_{\text{tot }}\rho }{\pi (1-\text{S}\rho )}.$$

## 4 Model intercomparison

As a first step for the RTM intercomparison study, we carried a global sensitivity analysis (GSA) of atmospheric RTM simulations. GSA allows us to identify the key input variables driving the spectral output and variables of lesser influence. By identifying variables of lesser influence, models and generated LUTs can be greatly simplified, which facilitates applications such as inversion of biophysical parameters and atmospheric correction. In short, sensitivity analysis algorithms determine the effect of changing the value of one or more input variables and observe the effect that this has on the RTM output. GSA, where the role of all input variables and their interactions are analyzed, has been successfully applied in vegetation and atmospheric RTMs (Verrelst et al.,2016; Vicent et al., 2017).

Here, we used ALG to generate a set simulations in order to analyze the relative impact of key atmospheric variables on TOA radiance. Three LUTs of MODTRAN5, libRadtran, and 6SV simulations were generated. They consist of 2000 samples distributed with a Latin hypercube sampling and cover the entire 400–2500 nm spectral range at 15 cm^–1^ (0.24–9nm) for MODTRAN and libRadtran and 2.5 nm for 6SV. These LUTs vary the atmospheric conditions as summarized in Table 1, with geometry fixed to SZA =30°, VZA =0°, and a relative azimuth angle (RAA) of 0°.

The generation of RT model input files is straightforward with ALG; the range of input variables given in Table 1 are introduced by the user through ALG’s interface. ALG processes this configuration and prepares the input files according to the user manual of each RT model for their specific format. For MODTRAN5 and libRadtran, all the input variables are actual parameters of these models as specified in the respective user manuals. For 6SV, the introduction of aerosol optical properties *α*, *G*, and SSA is achieved through the preparation of a specific 6SV .mie file. The reader should notice some of the main differences between the compared models as highlighted in Table 2 in order to support the later discussion about the observed differences. For all the 2000 combinations, the atmospheric transfer functions generated by ALG were coupled with a typical vegetation spectrum simulated with PROSAIL model (Jacquemoud et al., 2009) based on Eq. (2) using ARTMO’s TOC2TOA toolbox (Verrelst et al., 2019).

**Figure 4:**
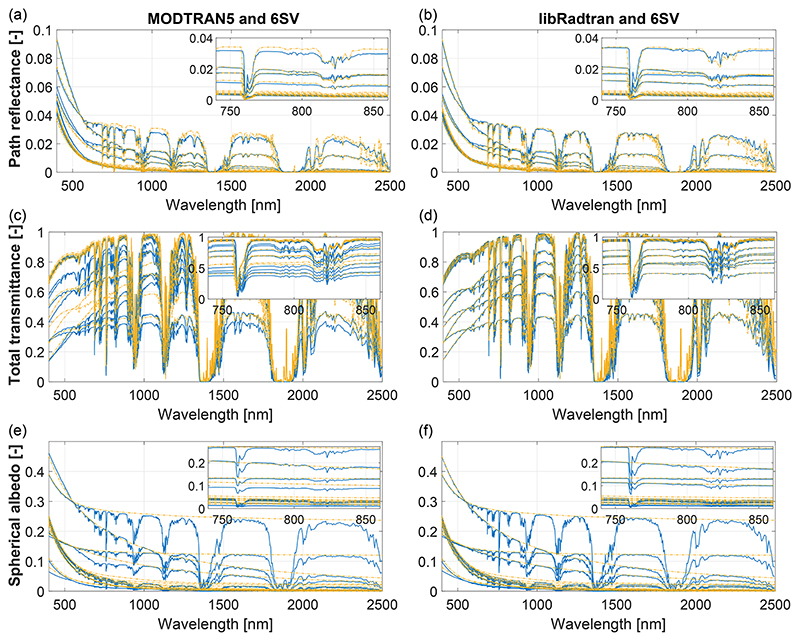
**(a, b)** Path reflectance, **(c, d)** total transmittance, and **(e, f)** spherical albedo spectra comparison between MODTRAN5 and libRadtran (blue, left and right columns, respectively) and 6SV (yellow).

Before analyzing the GSA results, we illustrate in Fig. 4 the path radiance, spherical albedo, and total solar irradiance calculated by the three selected atmospheric RTMs. In this figure only 16 spectra are shown, corresponding to all the min/max values of the four aerosol parameters given in Table 1 in order to illustrate the full variance in the database. The sub-axes zoom in the spectral window between 750 and 860 nm where the absorption features of the O_2_-A and H_2_O are visible.

This Fig. 4 illustrates the consistent MODTRAN, libRadtran, and 6SV simulations achieved with the use of ALG. Overall, it is observed how the three spectral magnitudes are overlapping in the entire 400–2500 nm spectral range. We can also observe that approximately six out of the 16 plotted spectra are mostly visible, which indicates that only two variables dominate the entire variance in the signal as it will later be discussed through the GSA analysis. Despite the agreement of the various RTMs, some discrepancies appear in the figures. Firstly, regardless of the spectral resolution, we find that 6SV has a better agreement with libRadtran than with MODTRAN5. The disagreement with MODTRAN5 is particularly higher at higher path reflectances and lower transmittances, which might indicate that MODTRAN tends to increase the effect of scattering through the phase function with respect to libRadtran and 6SV. Secondly, it is clearly observed how the spherical albedo in 6SV simulations is free of gas absorptions. Indeed, this is a result of the decoupling of gas absorption from scattering by molecules and aerosols in 6SV. Lastly, there are minor differences in the spectral features of the gas absorptions, which can be due to the absorption modelization (correlated-k in MODTRAN and Reptran in libRadtran) as well as differences in spectral resolution (2.5 nm in 6SV and 15 cm^–1^ in MODTRAN and libRadtran).

**Figure 5:**
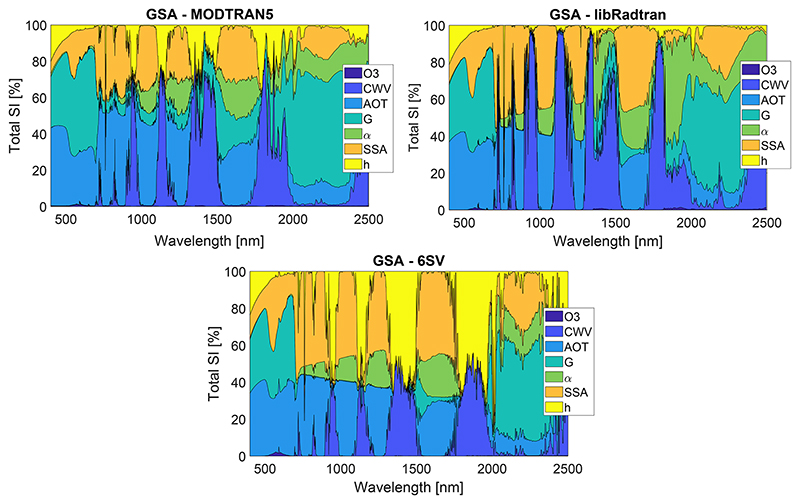
MODTRAN5, libRadtran, and 6SV GSA results of main atmospheric properties at TOA radiance.

The comparative analysis is followed in Fig. 5 through the total sensitivity index (SI), which shows the relative importance of each input variable at TOA radiance for typical vegetation spectrum.

In general, all three RTMs show similar GSA results, indicating that they simulate similarly the processes of absorption and scattering. In these models, the driving variables are those related to the aerosol particles (AOT, *α*, *G*, and SSA), which cause the scattering and thus path radiance and diffuse transmittance along the entire spectral range. The Ångström exponent increases its relative importance as wavelength increases from 550 nm, which is the anchor wavelength at which the AOT is defined. The surface altitude has its major influence (~ 80 %) at the bottom of the O_2_-A absorption (~ 760 nm) since the absorption is mostly driven by the surface pressure. As expected, the importance of CWV is localized at the specific wavelengths of H_2_O absorptions. All models also show a sudden decrease in the relative importance of the scattering processes (through the variables *G* and AOT) after ~ 720 nm. Indeed, according to Eq. (1), the high reflectance values of vegetation in the near infrared spectral region reduce the influence of the atmospheric path radiance (most affected by the scattering processes) with respect to the surface-reflected radiance. Despite of these similarities, the GSA figures also show some discrepancies, particularly on the lower importance of the aerosol absorption (through the SSA variable) in MODTRAN5 for wavelengths higher than ~ 720 nm. The MODTRAN5 model also shows some sensitivity (5 %–10 %) to the asymmetry parameter (*G*) in the 720–1300 nm spectral range, while it is nearly 0% in libRadtran and 6SV, in agreement with our observations in Fig. 4. Important differences also appear on the relative sensitivity of surface elevation and CWV within the H_2_O bands. In fact, both variables compete to influence the strength of the H_2_O absorption, the CWV through its influence on the amount of H_2_O in the atmospheric column and surface elevation directly by the definition of the optical path of photons. In this case, 6SV shows higher dependency on the surface elevation than MODTRAN and libRadtran due to uncoupled scattering and absorption effects in 6SV. In 6SV, the H_2_O absorption only affects to the direct Sun-target-sensor transmittance component, which is dependent on both the CWV and optical path (and thus surface elevation). In MODTRAN and libRadtran, the multiple scattering increases the optical path of photons and thus the absorption by H_2_O, which makes the model more sensitive to the CWV than surface elevation. However, MODTRAN and libRadtran still show differences in the relative sensitivity to CWV versus surface elevation, which indicates differences in the implementation of the coupled absorption–scattering processes at these strong absorption features or the definition of the scattering properties. In fact, the aerosol optical properties (i.e., *α*, *G*, and SSA) in MODTRAN are defined for the boundary layer aerosols, while in libRadtran and 6SV they are common for the entire column.

**Figure 6:**
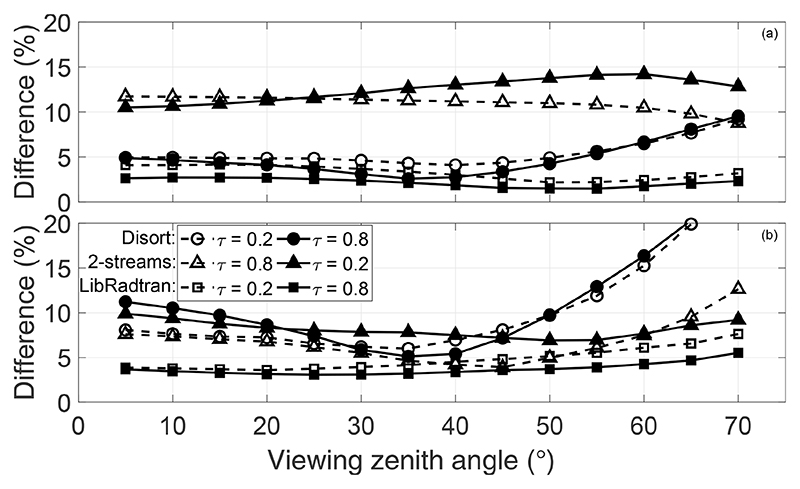
Average relative differences between 6SV and MODTRAN DISORT, Isaac’s 2-streams, and libRadtran DISORT for two wavelengths, **(a)** 412 nm and **(b)** 670 nm, as function of VZA and AOT.

To further prove the usefulness of ALG to perform RTM intercomparison, we secondly repeated the study in Kotchenova et al. (2008). Here, we compared 6SV simulations against MODTRAN’s DISORT (Stamnes et al., 1988) (eight streams) and Isaac’s 2-streams (Isaacs et al., 1987) RT solvers and libRadtran (DISORT solver with eight streams). The simulations were performed with a US Standard 1976 atmospheric profile, OPAC’s continental average aerosol model optical properties, two values of AOT (0.2 and 0.8), and the same range of illumination/observation conditions described in Kotchenova et al. (2008). The simulated LUTs were used to calculate the intrinsic atmospheric reflectance. The atmospheric reflectance from MODTRAN and libRadtran (*ρ*′) was compared with the simulated by 6SV ($\rho_{6\text{sv}}^{\prime }$) according to the following cost function:

(3) $$\delta (\tau ,\text{VZA},\lambda )=\frac{100}{N}\underset{\text{SZA}}{\sum^{\text{​}}}\underset{\text{RAA}}{\sum^{\text{​}}}\frac{\left|\rho_{6\text{sv}}^{\prime }-\rho^{\prime }\right|}{\rho_{6\text{sv}}^{\prime }},$$

where, for sake of simplicity, we have omitted the dependency of the reflectance on the AOT (τ), VZA, SZA, RAA, and wavelength (*λ*). Figure 6 shows the results of the average relative differences for two wavelengths (λ=412 and 670 nm).

The results are compatible with those presented in Kotchenova et al. (2008), showing differences (at 412 nm) of 5%–10% with respect to 6SV mostly due to the simulation of polarization in 6SV and the calculation of multiple-scattering by the Henyey–Greenstein aerosol phase function. These effects are also seen when using Isaac’s 2-streams radiative solver in MODTRAN, now with errors up to 15 %. The discrepancies with respect to libRadtran are rather constant, with errors around 3 %–4 %, probably since libRadtran introduces the phase function calculated by OPAC for the simulation of scattering effects.

## 5 Other applications

As described in the Sect. 3, ALG facilitates the usage of atmospheric RTMs and the generation of large LUTs of atmospheric transfer functions. Users can integrate these LUTs into a wide range of applications.

One of these applications is in end-to-end mission performance simulators (E2ES). E2ES are software tools that reproduce all aspects of satellite missions including the platform orbit/attitude, synthetic scene generation, sensor behavior, ground image processing, and product evaluation (Kerekes et al., 1999; Segl et al., 2012). These tools are used by remote sensing scientists and engineers to support trade-off studies, to prepare of system calibration tests, and to optimize data processing algorithms. As part of the European Space Agency FLEX E2ES (Vicent et al., 2016), precomputed MODTRAN-based LUTs generated with ALG are used to simulate the radiance signal as would be observed by FLEX mission instruments (Tenjo et al., 2017).

Another typical application of atmospheric LUTs is in the retrieval of aerosol physical and optical properties (Dubovik et al., 2002; Huang et al., 2015). In the context of satellite data processing, aerosols are one of the main atmospheric components that must be accounted for when performing atmospheric correction (Thompson et al., 2018). In this frame, we studied the impact of aerosol type variability in the atmospheric correction within the O_2_ absorption regions (Vicent et al., 2017). The goal was to determine whether parametric approximations in aerosol properties can be used to perform the atmospheric correction in the O_2_ absorptions. ALG was used to simulate several datasets with varying aerosol types, optical properties, and vertical distribution.

In addition to spaceborne instruments, ALG is also suitable for the analysis of airborne and proximal sensing (e.g., flux towers, unmanned aerial vehicles). In our publication Sabater et al. (2018), we studied the impact of path length in proximal sensing measurements of downwelling irradiance and at-sensor radiance and their impact on sun-induced fluorescence retrieval. The study focused on remote sensing instruments placed at 2–50 m height over the surface. ALG was used to facilitate the running of MODTRAN simulation, which varied the instrument height and the SZA for standard atmospheric conditions.

Altogether, these few examples demonstrate the versatility of ALG to address multiple remote sensing applications based on the use of atmospheric RTMs.

## 6 Conclusions and future work

In this paper the main design concept and features of ALG have been described along with an intercomparison study for the atmospheric RTMs 6SV, MODTRAN, and libRadtran. The a priori tedious tasks of (1) writing consistent input files, (2) running the RTMs in an efficient manner, (3) compiling and harmonizing the various model output files into ready-to-use LUT files, and (4) performing a model sensitivity analysis was largely simplified using the developed ALG tool and its compatibility with the ARTMO software framework (Verrelst et al., 2012). The sensitivity analysis results indicate that, overall, the various atmospheric RTMs simulate similarly the absorption and scattering processes for the selected atmospheric variables. However, there are still important differences in the sensitivity analysis that must be analyzed in more detail.

Other practical applications, such as scene generation, atmospheric data analysis, and atmospheric correction, can also benefit from the use of ALG. A few application examples were presented, demonstrating the software capabilities to generate consistent LUTs for several atmospheric RTMs, with a wide range of input atmospheric variables, nodes distribution, and spectral configurations. ALG is an ongoing work and regularly updated with new added functionalities and tools. The following upgrades are in the pipeline: (1) including the polarization data calculated by the 6SV code and the polRadtran and Mytic solvers in libRadtran, (2) implement functions to develop emulators of atmospheric transfer functions, (3) generation of LUTs of TOA radiance spectra for non-Lambertian and nonhomogeneous surfaces, (4) implementation of additional RTMs such as RRTOV and SOS, and (5) compatibility with Linux and MacOS systems. In summary, ALG can become an useful tool to facilitate research on atmospheric radiative transfer, as well as opening the use of atmospheric RTMs to wider research communities and applications such as for climate studies, atmospheric physics and chemistry, and remote sensing data processing.

## Figures and Tables

**Table 1:** Key input atmospheric variables used in MODTRAN5, libRadtran, and 6SV to perform the GSA. Atmospheric profile was set to US Standard 1962 (Anderson et al., 1986).

Variable name	Min–max
Elevation (*h*):	0–3 km
Aerosol optical thickness (AOT):	0.05–1
Ångström exponent (*α*):	0.1–1.5
Asymmetry parameter (*G*):	0.6–1
Single scattering albedo (SSA):	0.75–1
Water vapor (CWV):	1–4 g cm^2^
Ozone (O_3_):	0.25–0.45 atm-cm

**Table 2:** Key commonalities and differences between MODTRAN5, libRadtran and 6SV simulations.

Feature	MODTRAN5	libRadtran	6SV
RT solver:	DISORT	DISORT	Successive orders of scattering
Absorption modelization (resolution):	Correlated-*k* (15 cm^–1^)	Reptran (15 cm^–1^)	Band model (2.5 nm)
Coupled absorption-scattering (yes/no):	Yes	Yes	No
Aerosol optical prop. (input config.):	Input parameters (i.e., *α*, *G,* SSA)		Precalculated through .mie file
Aerosol optical prop. (vertical distr.):	Only in boundary layer	Optical properties common for entire column	

## Data Availability

The exact version of the ALG (v2.0) used to produce the results used in this paper is archived on Zenodo (https://doi.org/10.5281/zenodo.3555575, Vincent et al., 2019), as are input data and scripts to run the model and produce the plots for all the simulations presented in this paper. The current version of ALG is freely available from the project website (https://artmotoolbox.com/, last access: 15 April 2020; Verreslt and Rivera-Caicedo, 2020) under the GNU General Public License v3 (see http://www.gnu.org/licenses/, last access: 15 April 2020; Free Software Foundation, Inc, 2018). The software package has been developed in MATLAB^®^ R2018a, and it is compatible with Windows operating systems. The tool is also provided as a stand-alone compiled executable file so that users not having a license of MATLAB can still run the software. Accordingly, users must first install the corresponding MATLAB Runtime (MCR version 9.5, 64 bits). In addition, the help system of ALG includes a set of guidelines to install and compile the compatible atmospheric RTMs. The user should notice that ALG does not redistribute the source code or the compiled version of the underlying third-party atmospheric RTMs due to license rights.
